# Population-Based Prevalence of Ocular *Chlamydia trachomatis* Infection among Infants in the Trachoma Endemic Amhara Region, Ethiopia

**DOI:** 10.4269/ajtmh.21-0873

**Published:** 2021-10-25

**Authors:** Scott D. Nash, Ambahun Chernet, Tigist Astale, Eshetu Sata, Mulat Zerihun, Andrew W. Nute, Kimberly A. Jensen, Demelash Gessese, Zebene Ayele, Berhanu Melak, Mahteme Haile, Taye Zeru, Zerihun Tadesse, E. Kelly Callahan

**Affiliations:** ^1^ Trachoma Control Program, The Carter Center, Atlanta, Georgia;; ^2^ Trachoma Control Program, The Carter Center, Addis Ababa, Ethiopia;; ^3^ Research and Technology Transfer Directorate, Amhara Public Health Institute, Bahir Dar, Ethiopia

## Abstract

Infants ages < 6 months do not receive azithromycin as part of trachoma control and thus may serve as an infection reservoir in persistently endemic districts. The aim of this study was to determine the population-based *Chlamydia trachomatis* infection prevalence and infectious load among infants ages 1–12 months in persistently trachoma endemic districts in Amhara, Ethiopia. Across six districts, 475 infants were enumerated, and of these 464 (97.7%) were swabbed for infection testing. The *C. trachomatis* infection prevalence in the study area among infants was 0.2% (95% CI: 0.0–1.5). Among children ages 0–5 years positive for *C. trachomatis*, the median load was 31 elementary bodies (EB) (Inter quartile range: 7–244 EB), and the infection-positive infant had a load of 7,755 EB. While it is worth reconsidering azithromycin treatment recommendations for the potential mortality benefits, these results do not support lowering the treatment age for trachoma control.

## INTRODUCTION

Some trachoma-endemic districts in Amhara region, Ethiopia, have received ≥ 10 annual rounds of antibiotic mass drug administration (MDA) with > 80% reported population coverage and still have a persistently high prevalence of trachoma and its causative agent, ocular *Chlamydia trachomatis* infection.^1^^,^^2^ Trachoma MDA programs deliver azithromycin to individuals ages ≥ 6 months and tetracycline eye ointment (TEO), twice daily for 6 weeks, to individuals age < 6 months. Although TEO has been shown to be effective against *C. trachomatis*, it has been demonstrated that compliance is poor.^3^^–^^5^ Infants could represent a potential infection reservoir within communities, as many do not receive azithromycin under current treatment guidelines. Furthermore, infants may be missing a potential mortality benefit, as azithromycin MDA has shown to reduce childhood mortality.^6^ The aim of this study was to determine the *C. trachomatis* infection prevalence and infectious load among a population-based sample of infants ages 1–12 months in districts experiencing persistently endemic trachoma in Amhara.

## METHODS

Survey methodology was approved by the Amhara Regional Health Bureau, Emory University under protocol 079-2006, and reviewed by Tropical Data (https://www.tropicaldata.org/). Because of the high illiteracy rate among the population, Internal Review Board approval was obtained for oral consent or assent for older children. Oral consent or assent was obtained and recorded electronically for all individual participants according to the Declaration of Helsinki.

This study was conducted in six districts (Supplemental Figure 1) of East Gojjam zone, Amhara with hyperendemic (> 30%) trachomatous inflammation-follicular (TF) as of 2012 after approximately 5 years of SAFE interventions.^7^ Since 2012, each of these districts has received 5 additional years of interventions.^1^ As these districts are geographically adjacent, and have similar epidemiology and treatment history, they were combined into one enumeration unit (EU).

Between September and October 2018, approximately 8 months since the last MDA, population-based trachoma impact surveys were conducted in these six districts using well-characterized methods.^1^^,^^7^^,^^8^ Briefly, two-stage probability sampling was used whereby 30 clusters were selected using a probability proportional to estimated size method in the first stage, and segments of 30 households were randomly selected from each cluster in the second stage.^1^ To determine the EU prevalence of *C. trachomatis* among infants, 410 infants 1–12 months were targeted for swabbing, which assumed a prevalence of 3% ± 3% based on earlier data from the region: α error of 0.05, design effect of 3.0, and 10% nonresponse rate.^9^ Based on previous surveys, infants make up 1.9% of the population; therefore, approximately 70 infants per district and 420 per EU were expected.

All individuals > 1 year were graded for the WHO simplified signs TF, trachomatous inflammation-intense (TI), and trachomatous trichiasis by a certified grader using a ×2.5 loupe and adequate light.^10^ For this study specifically, all children ages 0–5 years residing in selected households provided an ocular swab.

After trachoma grading, the gloved grader passed a polyester tipped swab across the conjunctiva firmly three times, rotating 120° between each pass.^2^^,^^9^ Swabs were then placed into 2 mL vials without preservative, stored on ice in the field, and then at −20°C until assaying at the Amhara Public Health Institute.

Swabs were randomized by district, pooled five swabs per pool, and assayed with the Abbott RealTime (Abbott Molecular Inc., Des Plaines, IL) polymerase chain reaction assay for *C. trachomatis*, using the Abbott m2000 system between January and June 2019.^2^^,^^9^ To generate individual results, individual samples from positive pools were assayed again, and all individuals from negative pools were considered negative.^9^ Prior to individual-level testing, a calibration curve was created using a standard set of elementary body (EB) titrations.^9^^,^^11^ Laboratory quality control procedures have been detailed previously.^2^^,^^9^

Prevalence of TF, TI, and *C. trachomatis* infection was calculated as the mean of each cluster prevalence after cluster-level age-adjustment (1year age bands) using the national census data.^8^ Confidence intervals (CI) were calculated using a bootstrap method.^8^ Taylor linearization was used to estimate CIs for age-specific results, accounting for household and cluster level clustering.^1^ Logistic regression was used to test for associations between age, sex, and trachoma outcomes while controlling for household and cluster level clustering.

## RESULTS

Across the six-district EU, 464 infants (97.7%) were swabbed out of 475 infants enumerated. Infants made up 2.2% of the EU population. A further 4,608 children ages 1–9 years were examined for trachoma signs, 2,302 children ages 1–5 years were also swabbed.

The district-level TF prevalence among children ages 1–9 years ranged from 4.1% to 23.4%, and the prevalence of *C. trachomatis* infection among children ages 1–5 years ranged from 0.0% to 4.0% (Supplemental Table 1). The *C. trachomatis* infection prevalence in the EU among infants was 0.2% (95% CI: 0.0–1.5). The single infection-positive infant was 10 months old and swabbed in Shebel district.

**Figure 1.**
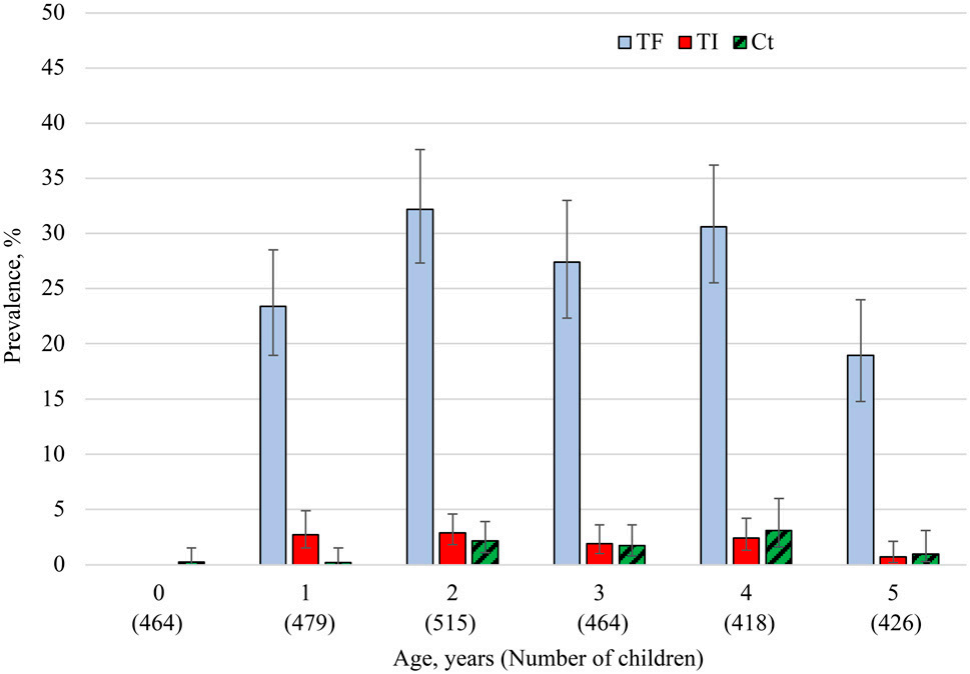
Prevalence of TF, TI, and *C. trachomatis* infection by age 0–5 years, East Gojjam zone Amhara, Ethiopia, 2018. This figure appears in color at www.ajtmh.org.

**Figure 2.**
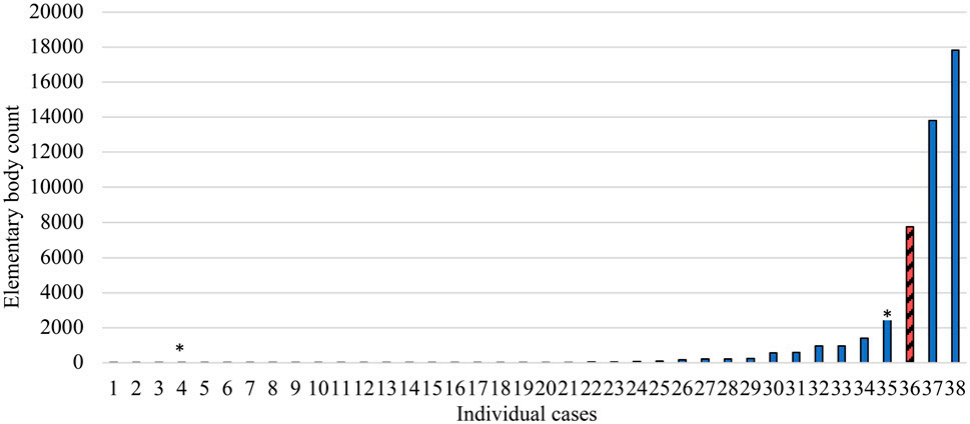
*C. trachomatis* load among 38 children ages 0–5 years positive for ocular *C. trachomatis,* East Gojjam zone, Amhara, Ethiopia, 2018. Red hatched bar indicates the *C. trachomatis* load for the single infected infant. *Individuals positive for *C. trachomatis* infection living in neighboring households to the infected infant. This figure appears in color at www.ajtmh.org.

*Chlamydia trachomatis* infection prevalence among children ages 0–5 years was lowest among infants (0.2%) and highest among children ages 4 years, 3.1% (95% CI: 1.6–6.0) (Figure 1). *Chlamydia trachomatis* infection prevalence increased with age (*P* = 0.01) but did not differ by sex (*P* = 0.14) in this age group. Among swabbed children TF prevalence increased with age (*P* < 0.001) and was higher in boys than girls (24.0% versus 20.6%, *P* = 0.03), while TI prevalence did not differ by age or sex (*P* = 0.47; *P* = 0.11). For each age, the prevalence of TF was considerably higher than that of *C. trachomatis* and TI. Among infection-positive children ages 0–5 years, the median load was 31 EBs (Inter quartile range: 7–244 EBs). The infection-positive infant had the third highest load of 7,755 EBs (Figure 2).

**Figure 3.**
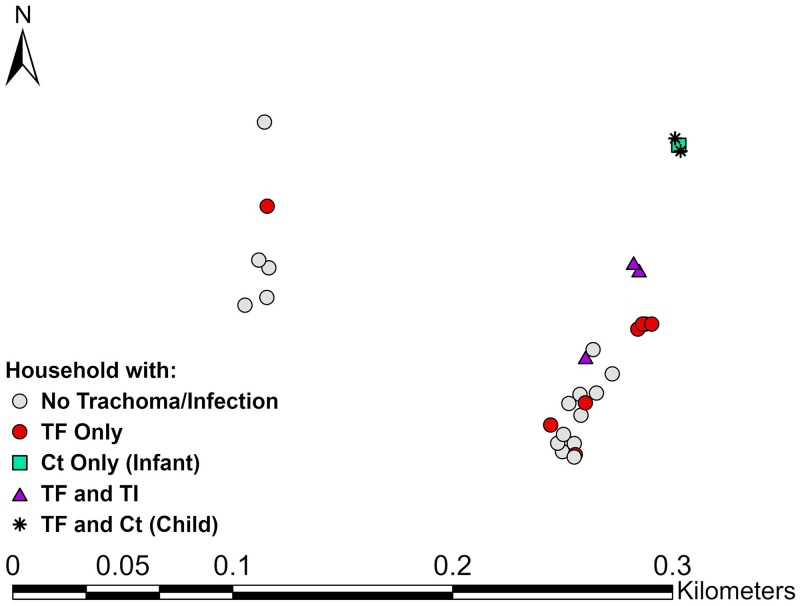
Spatial distribution of trachoma outcomes within the cluster where the infected infant resided, East Gojjam zone, Amhara, Ethiopia, 2018. This figure appears in color at www.ajtmh.org.

Sixty infants (12.9%) were swabbed in households where a family member had clinical signs (range 1–4 members), and one infant resided with an infected child; however, none of these infants were *C. trachomatis* positive. While the *C. trachomatis* positive infant lived in a household where no other family members were infected or had clinical signs, the infant lived close to two *C. trachomatis* positive children in a cluster with 10.5% infection prevalence (Figure 3).

## DISCUSSION

Within a persistently trachoma endemic area of Amhara, infants were likely not a considerable community *C. trachomatis* reservoir. Nearly all infants were *C. trachomatis* negative, even though the majority were too young to be eligible for azithromycin at the MDA 8 months prior to the survey. One infected infant with a high chlamydial load lived in a community with a considerable trachoma burden, suggesting that infection among infants may be a larger concern in hyperendemic settings.

In previous village-level studies in highly trachoma endemic settings outside of Ethiopia, considerable *C. trachomatis* infection was detected among infants prior to MDA, and infectious load among infants was greater than or equal to that of older children.^12^^–^^15^ The long-running MDA program in Amhara may have had protective effects for infants in this study area as indirect benefits from MDA have been previously demonstrated among young children in Ethiopia, or TEO treatments may have been more effective than commonly assumed.^16^

Mass drug administration with azithromycin has ancillary benefits for the recipient communities.^6^^,^^17^ Recent randomized control trial results demonstrated a beneficial mortality effect with azithromycin MDA, observed most strongly among children ages < 6 months.^6^ Current WHO recommendations specify treating children ages < 6 months with TEO instead of azithromycin. While it is worth reconsidering this recommendation for mortality benefits, our results do not support lowering the eligible azithromycin treatment age for trachoma control alone.

Estimating *C. trachomatis* infection prevalence among infants within a programmatic setting was possible with a high response rate. This study was conducted alongside trachoma impact surveys which limited the data collection to cross-sectional assessments of trachoma simplified signs and conjunctival swabbing. More frequent longitudinal monitoring of infants and children of all ages with swabbing and finer trachoma grading scales would help to better understand transmission dynamics within persistently endemic communities. A limitation was not assessing clinical signs among infants. Due to the developing nature of the immune system, however, it is believed that clinical signs are difficult to assess among infants, particularly those ages < 6 months.^15^^,^^18^ Furthermore, knowing infection status is more informative to programs in gauging the potential of this age group in serving as an infection reservoir. While the infection assay could not discriminate between ocular and genital Chlamydial strains, the local epidemiology suggested a likelihood that ocular *C. trachomatis* transmission was occurring in the infected infant’s community. Given that infants are likely not an infection reservoir, other contributors to the persistent nature of trachoma experienced by some countries should be investigated if elimination of trachoma as a public health problem is to be achieved by 2030.

## Supplemental tables


Supplemental materials
