# Supplementary material for: Population-Based Prevalence of Ocular Chlamydia trachomatis Infection among Infants in the Trachoma Endemic Amhara Region, Ethiopia
Source: Am J Trop Med Hyg. 2021 Oct 25;106(1):62–5. doi: 10.4269/ajtmh.21-0873 (PMC8733485; doi:10.4269/ajtmh.21-0873)
Supplement: Supplementary file 1 [file tpmd210873.SD1.pdf]

Supplemental Figure 1. Study location and district level trachomatous inflammation-follicular (TF) prevalence among children ages 1-9 years, Amhara, Ethiopia, 2018

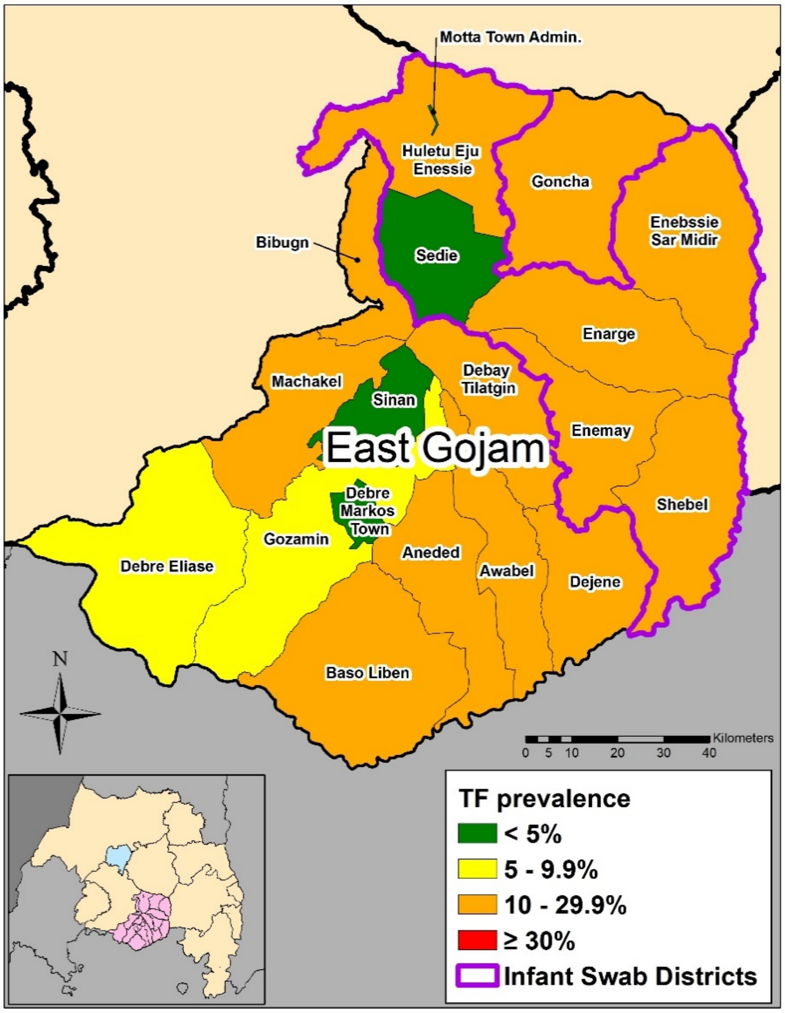

Supplemental Table 1. Trachoma prevalence data for districts included in the study enumeration unit, Amhara, Ethiopia, 2018

District	N, ages 1-9 y	TF 1-9 y, % (95%CI)	TI 1-9 y, % (95%CI)	N, ages 1-5 y	*Ct* 1-5y, % (95%CI)
Enarge	589	16.9% (12.5-23.3)	2.5% (1.1-4.0)	272	1.1% (0.0-2.5)
Enebssie Sar Midir	808	16.2% (11.7-22.0)	1.0% (0.4-1.8)	405	0.3% (0.0-1.0)
Enemay	647	23.4% (17.8-29.7)	4.0% (1.8-6.6)	344	3.8% (1.3-6.9)
Huletu Eju Enessie	958	13.4% (10.0-17.7)	0.8% (0.3-1.3)	490	1.3% (0.0-3.2)
Sedie	863	4.1% (2.0-6.8)	0.4% (0.1-0.8)	461	0.0% (0.0-0.0)
Shebel	743	22.9% (16.8-27.7)	1.6% (0.6-3.0)	331	4.0% (1.4-7.3)

TF = trachomatous inflammation-follicular; TI = trachomatous inflammation-intense; *Ct* = *Chlamydia trachomatis*; y = years; 95% CI = 95% confidence intervals.
